# Clinicopathologic Features and Prognosis of *BRAF* Mutated Colorectal Cancer Patients

**DOI:** 10.3389/fonc.2020.563407

**Published:** 2020-11-23

**Authors:** Wen-Long Guan, Miao-Zhen Qiu, Cai-Yun He, Li-Qiong Yang, Ying Jin, Zhi-Qiang Wang, Yu-Hong Li, Rui-Hua Xu, Feng-Hua Wang

**Affiliations:** ^1^State Key Laboratory of Oncology in South China, Department of Medical Oncology, Sun Yat-sen University Cancer Center, Collaborative Innovation Center for Cancer Medicine, Guangzhou, China; ^2^State Key Laboratory of Oncology in South China, Department of Molecular Diagnostics, Sun Yat-sen University Cancer Center, Collaborative Innovation Center for Cancer Medicine, Guangzhou, China; ^3^State Key Laboratory of Oncology in South China, Department of Experimental Research, Sun Yat-sen University Cancer Center, Collaborative Innovation Center for Cancer Medicine, Guangzhou, China

**Keywords:** *BRAF*, V600E, CDX2, colorectal cancer, prognosis

## Abstract

**Background:** *BRAF*^V600E^ mutation is associated with poor prognosis of colorectal cancer (CRC) patients, but the comparison of clinic-pathologic features between V600E and non-V600E mutation was not well-known in CRC patients. The aim of this study is to evaluate the clinical and pathological features, prognostic value of *BRAF* mutations in CRC.

**Methods:** We conducted a retrospective study to characterize the clinical and pathological features and survival of patients with *BRAF* mutated CRC. Patients were classified according to *BRAF* status as *BRAF*^V600E^ mutation and non-V600E mutations. Difference of characteristics and survival between the two groups was analyzed.

**Results:** There was no significant difference in gender, family history, location of primary tumor, metastatic sites between patients with *BRAF*-V600E mutation and non-V600E mutations. Patients with V600E mutation were younger than those with non-V600E mutations (*p* = 0.002). Patients with *BRAF*^V600E^ mutation showed a poorer outcome than those with non-V600E mutations (23.1 vs. 49.9 months, respectively, *p* = 0.0024). Lack of CDX2 expression was associated with worse prognosis (mOS: 9.4 m vs. not reached, respectively, *p* = 0.016). Status of V600E mutation did not affect the mPFS and ORR of first-line or second-line treatment.

**Conclusion:** *BRAF*^V600E^ mutation defines a distinct subgroup of CRC with worse prognosis. Lack of CDX2 expression is associated with poor OS. Status of V600E mutation did not affect the mPFS of first-line or second-line treatment.

## Introduction

Colorectal cancer (CRC) is the third most prevalent malignancy worldwide (1). CRC is widely recognized as a molecularly heterogeneous disease, resulted from accumulation of genetic and/or epigenetic changes involving several pathways, such as microsatellite instability (MSI), chromosomal instability (CIN), RAS-RAF-MEK-ERK-MAPK pathway. Among them, mutations in *RAS* and *BRAF* (v-raf murine sarcoma viral oncogene homolog B) genes are most widely used in clinical decision making (2). *BRAF*, a proto-oncogene, plays an important role in cell differentiation, proliferation and survival through MAPK pathway (3). Therefore, its aberrant activation is critical for tumorigenesis in many types of malignancies, such as melanoma, hairy cell leukemia, papillary thyroid carcinoma, non-small cell lung cancer (NSCLC) as well as CRC (4–9). In CRC, the incidence of *BRAF* mutation is about 3–10% (4, 10–12). The most common *BRAF* mutation is due to a CTGCAG change in the nucleotide 1,799 of exon 15 (T1799A), which leads to an amino acid substitution from valine to glutamate at codon 600 (p.V600E). This mutation is known as *BRAF*^V600E^ mutation, which accounts for 56–90% of *BRAF* mutations (13–16). Many studies have demonstrated the negative prognostic value of *BRAF* V600E mutation on metastatic CRC patients (4, 8). However, in our clinical practice, we found that not all the *BRAF*^V600E^ patients had poor prognosis. Moreover, non-V600E *BRAF* mutations are less common in CRC, and their clinical and pathological features, prognostic and predictive value were less discussed.

Since the behavior of *BRAF*^V600E^ mutated mCRC is aggressive, the PFS of traditional chemotherapy is poor and only 60% of patients can receive second-line treatment. Hence, intensive combination of targeted therapy and chemotherapy may be effective. FOLFOXIRI plus bevacizumab regimen has demonstrated an improved PFS and OS (17). So, it is recommended during first-line treatment for *BRAF*^V600E^ mutated mCRC. During second-line treatment, combined approach with several targeted inhibitors against different key components of MAPK pathway has showed promising results, with a median progression free survival (PFS) of 7.7 months by vemurafenib, irinotecan, and cetuximab (18, 19), or 8.0 months by encorafenib, binimetinib, and cetuximab (20). To our knowledge, there are no studies about effectiveness of chemotherapy for Chinese CRC patients with BRAF mutation. In this study, we evaluated these mutations and tried to provide new insights of Chinese *BRAF* mutations CRC patients.

## Methods

### Clinical Data

In this study, we retrospectively review CRC patients with BRAF mutation who were diagnosed between April 2013 to January 2020 at Sun Yat-sen University Cancer Center (Guangzhou, China). All the patients were diagnosed as CRC by hematoxylin and eosin (HE) staining and histologically analysis. Clinic records, including gender, age, primary tumor location, TNM stage at diagnosis, metastatic sites, family history, MSI/MMR status, date of diagnosis and date of last contact, were collected by our medical record system.

### Ethics and Consent Statement

The studies involving human participants were reviewed and approved by ethics committee of Sun Yat-sen University Cancer Center. The patients provided written informed consent to participate in this study.

### DNA Extraction, and NGS Library Preparation and Sequencing

DNA from the tumor tissues and their paired normal tissues or peripheral blood cells were extracted using the QIAamp DNA FFPE Tissue kit (Qiagen, Hilden, Germany) according to the protocols recommended by the manufacturer as previously described (21). DNA concentration was measured using the Qubit dsDNA HS Assay kit on a Qubit Fluorometer 3.0 (Life Technologies, Carlsbad, CA, USA). Gene mutations of samples collected before February 2019 were tested by the OncoCarta Panel version 1.0 (Sequenom Inc., San Diego, CA, USA) which covered a total of 238 possible mutations in 19 common oncogenes as previously described (12). OncoScreen Panel (Burning Rock Biotech Ltd, Guangdong, China) was used for detection of 295 key genes since February 2019. The threshold of input DNA quantity was 200 ng for samples to proceed to library preparation, as previously described (22, 23). Fragments between 200 and 400 bp were purified by AGEcout AMPure beads (Beckman Coulter, Pasadena, USA). Hybridization, hybrid selection and PCR amplification were then performed according to the commercial protocol, and the indexed samples were sequenced on an Illumina NextSeq500 sequencer with pair-end reads (Illumina, Inc., San Diego, USA). A minimal median unique sequencing depth of 500X was necessary and sufficient to assess low frequency mutations for each tumor sample.

### Statistical Methods

The patients' clinicopathological features were summarized with descriptive statistics. Categorical variables were compared using Chi square test, and comparisons of continuous variables were performed using Student's *t*-test. Five-year cause specific survival (CSS) was calculated from the date of diagnosis to the date of cancer-specific death. Survival among different variables was compared using Kaplan-Meier estimates and the log-rank test. Statistical analysis was carried out by the IBM SPSS Statistics 22.0.0 package software (SPSS Inc) and the Intercooled Stata 13.0 (Stata Corporation, College Station, TX). All the *P*-values were two-sided, and statistical significance was set at *P* < 0.05.

## Results

### Patients Characteristics

From April 2013 to January 2020, 74 Chinese CRC patients with *BRAF* mutations were investigated in Sun Yat-sen University Cancer Center. Fifty four (73.0%) were *BRAF*^V600E^ mutated. Most patients were diagnosed at advanced stage (59/74, 79.7% at stage IV). There were 26 (35.1%) right-sided (cecum to transverse colon) and 19 (25.7%) left-sided (splenic flexure to sigmoid colon) cases, and the rest were in rectum (29/74, 39.2%). Patients with V600E mutation were much younger than those with non-V600E mutations (48.1 vs. 58.8 years old, *p* = 0.002). The most common sites of non V600E mutations are codon 469, 464, and 594. There was no significant difference in gender, family history, location of primary tumor, metastatic sites, CDX2 status, MSI status or TMB level between V600E and non-V600E groups. Though RAS and BRAF genes were thought to be mutually exclusive, 4 cases with RAS and BRAF co-mutations were found in our study. All of them were non-V600E mutated. The clinical and pathological features are showed in Table 1.

**Table 1:** Clinical characteristics of colorectal cancer patients with *BRAF* mutation.

	**V600E *N* = 54**	**Non-V600E *N* = 20**	***P*-value**
Gender			0.653
Female	22 (40.7)	7 (35.0)	
Male	32 (59.3)	13 (65.0)	
Age			0.002
Mean ± SD	48.1 ± 13.1	58.8 ± 11.2	
Median	48	63	
Family history			0.566
No	39	16	
Lung cancer history	8	3	
Colorectal cancer history	2	1	
Other cancer history	5	0	
Location			0.076
Right-sided colon	22	4	
Left-sided colon	15	4	
Rectum	17	12	
RAS			0.001
Wild type	54	16	
Mutation	0	4	
PI3K			0.348
Wild type	46	14	
Mutation	8	6	
MSI status			0.401
MSS/MSI-L	44	12	
MSI-H	1	1	
Unknown	9	7	
TMB			0.440
Mean ± SD	7.3 ± 3.6	8.4 ± 1.7	
Median	7.1	8.2	
CDX2			0.453
Positive	21	7	
Negative	7	1	
Unknown	26	12	
TNM stage			0.355
I	0	1	
II	2	1	
III	7	4	
IV	45	14	
Metastasis site			
Liver	30	12	0.732
Lung	13	8	0.177
Peritoneal	23	7	0.555
Bone	1	3	0.058
Distant lymph node	20	6	0.685

Eight patients with negative CDX2 expression were found in our study. The median age was 47.3 (30–69) years. Most of them (6/8, 75%) were male. Seven (87.5%) of them were *BRAF*^V600E^ mutated. In terms of primary tumor location, there were 3 cases on each side of colon, and the rest 2 cases were located in the rectum. No remarkable difference of age, gender, location, differentiation, metastatic site was found in patients with negative CDX2 compared to those with positive CDX2 expression.

### Treatment

**Figure 1:**
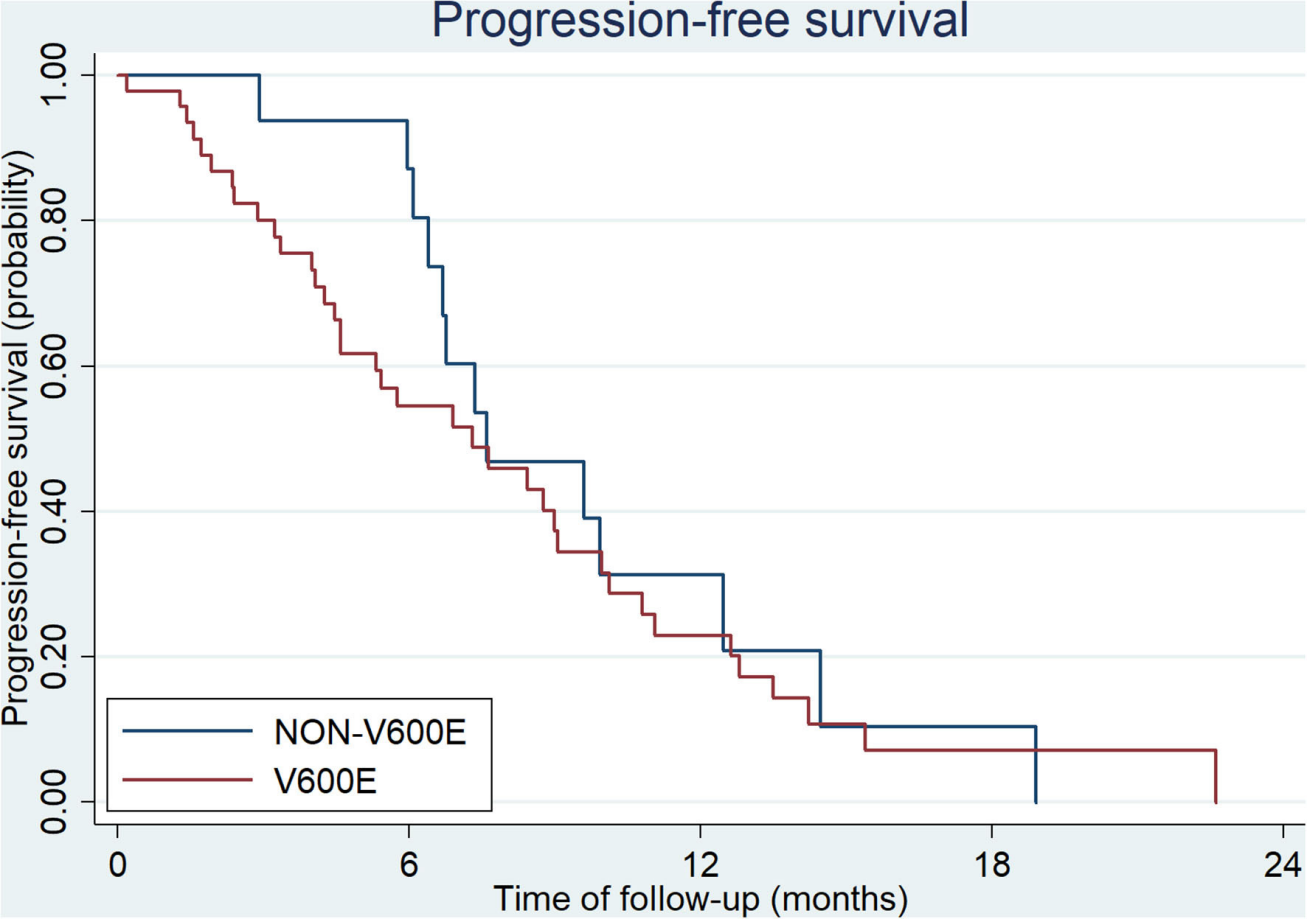
First-line PFS of CRC patients with BRAF V600E and non-V600E mutation (ECOG = 0–2).

All patients at stage I, II, and III (9/54 in V600E group and 6/20 in non-V600E group, respectively), received radical surgery. Sixty five patients received first-line therapy and 58 of them were evaluable. Forty eight patients with *BRAF*^V600E^ mutation received first-line treatment, and the regimen mostly used was bevacizumab plus two or three-drug chemotherapy (Table 2). Besides, 2 patients received local therapies for primary tumor and metastatic sites in the condition of disease controlled after systemic treatment. Among the 20 cases with *BRAF*^non−V600E^ mutation, 17 patients received first-line treatment, including 10 cases with chemotherapy alone, 5 with chemotherapy plus cetuximab, and 2 with chemotherapy plus bevacizumab. Two patients received local therapies for liver/lung metastasis. Median progression free survival (mPFS) and objective response rate (ORR) of patients with *BRAF*^V600E^ mutation was 7.3 months (95%CI: 4.6–9.1 months) and 30.1%, while patients with non-V600E mutations had an mPFS of 7.6 months (95%CI: 6.4–12.5 months) and an ORR of 37.5%. PFS and ORR during first-line treatment was not affected by status of V600E mutation (*p* = 0.51 and 0.64, respectively, Figure 1). PFS of different regimens for *BRAF*^V600E^ mutated patients is showed in Table 2. It seems that regimens with Bevacizumab + chemotherapy had a better PFS than chemotherapy alone or chemotherapy plus cetuximab, but the statistical significance was not reached.

**Table 2:** First line therapy for patients with *BRAF*^V600E^ mutated colorectal cancer.

**Regimen**	**Partial response**	**Stable disease**	**Progression disease**	**mPFS (months)**
Bevacizumab + FOLFOXIRI (*N* = 11)	6	3	2	8.8
Bevacizumab + FOLFOX/FOLFIRI/XELOX (*N* = 9)	2	5	2	9.1
FOLFOXIRI/FOLFOX/FOLFIRI/XELOX (*N* = 19)	4	8	7	4.6
Cetuximab + FOLFOX/FOLFIRI (*N* = 2)	0	2	0	4.3

**Figure 2:**
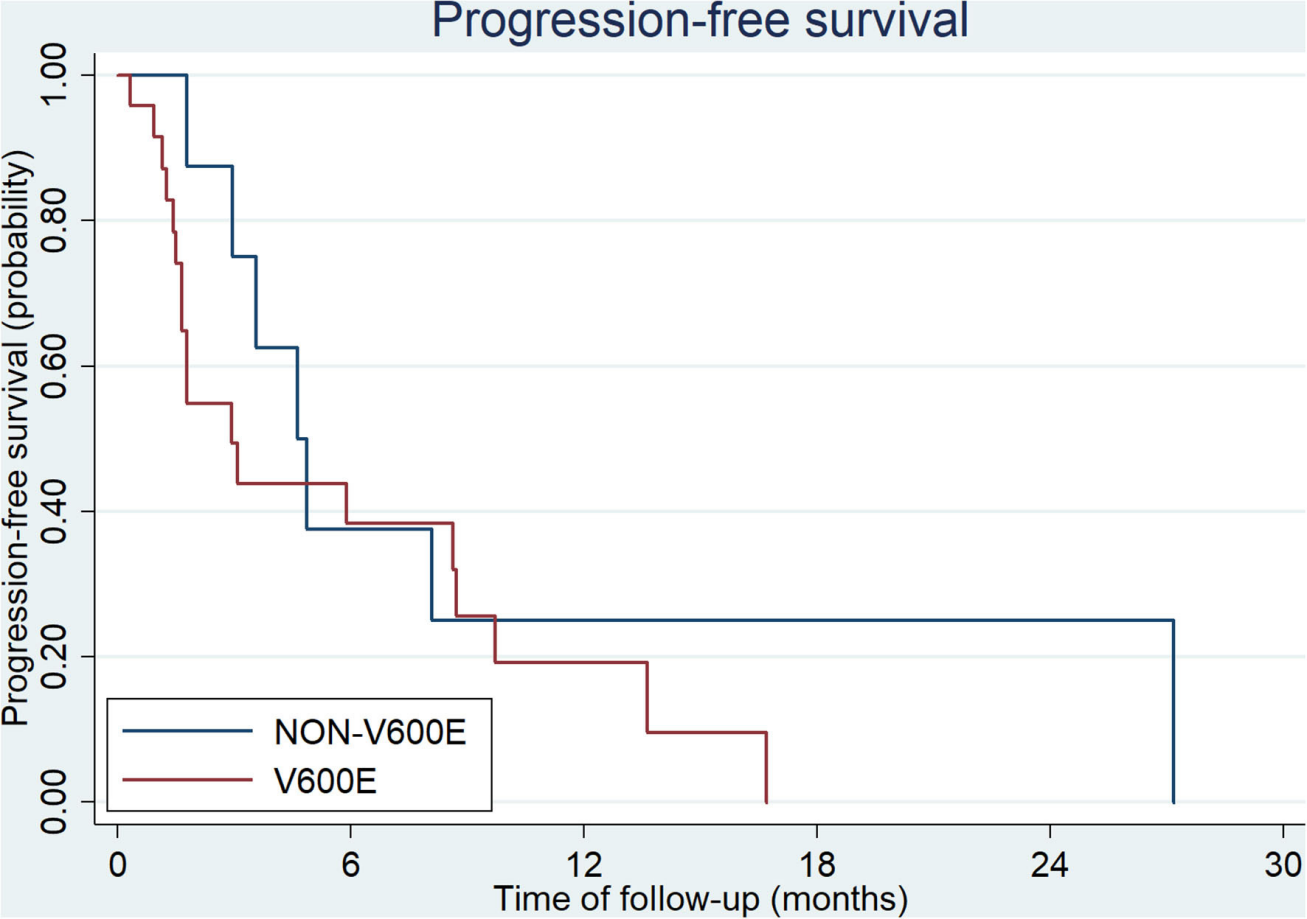
Second-line PFS of CRC patients with BRAF V600E and non-V600E mutation (ECOG = 0–2).

Thirty three patients received second-line therapy. the regimens mostly used for patients with *BRAF*^V600E^ mutation were VIC (vemurafenib, irinotecan and cetuximab) and bevacizumab plus chemotherapy (Table 3). Among patients with *BRAF*^non−V600E^ mutation, only 8 patients (8/17, 47%) received second-line treatment. Four of them received chemotherapy alone, 3 received bevacizumab plus chemotherapy, and 1 patient received cetuximab plus chemotherapy. mPFS for patients with *BRAF* ^V600E^ and non-V600E mutations was 2.9 months (95%CI: 1.7–8.7 months) and 4.6 months (95%CI: 1.8- months), respectively (*p* = 0.30, Figure 2). The ORR of *BRAF* ^V600E^ and non-V600E mutations was 14.3 and 12.5%, respectively, *p* = 0.90. The effect of different regimens for patients with *BRAF*^V600E^ mutation is presented in Table 3. Bevacizumab + chemotherapy seemed to have an improved PFS (9.7 months) compared to other regimens, though it was not statistically different.

**Table 3:** Second line chemotherapy for patients with *BRAF*^V600E^ mutated colorectal cancer.

**Regimen**	**Partial response**	**Stable disease**	**Progression disease**	**mPFS (months)**
VIC (*N* = 8)	1	5	2	2.9
Bevacizumab + FOLFOXIRI/FOLFIRI (*N* = 8)	1	4	3	9.7
FOLFOXIRI/FOLFIRI (*N* = 2)	0	0	2	1.2
Regorafenib/Fruquintinib (*N* = 3)	1	1	1	1.8

Among the 8 patients with loss of CDX2 expression, 6 patients received first-line treatment. Two of them were treated with bevacizumab plus chemotherapy, and the other 4 patients used chemotherapy alone. The ORR was 16.7% (1/6) and mPFS was 3.2 months.

### Survival Analysis

**Figure 3:**
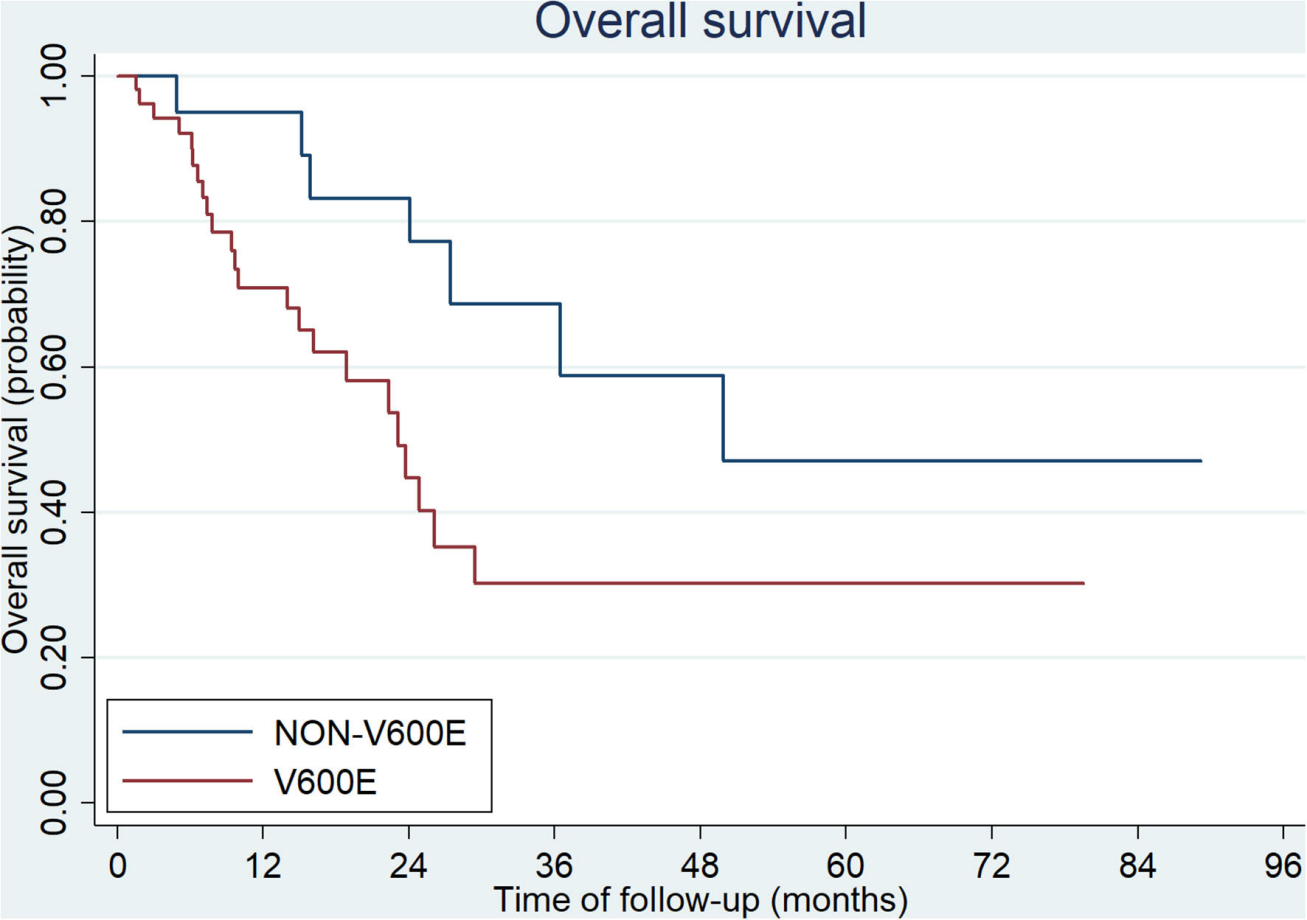
Overall survival of CRC patients with BRAF V600E and non-V600E mutation.

The median overall survival (OS) of all patients was 27.4 months in our study. Patients with *BRAF*^V600E^ mutation showed a poorer outcome than those with non-V600E mutations (23.1 vs. 49.9 months, *p* = 0.0024, Figure 3). There were 15 patients diagnosed at early stage (stage I, II, and III; 9/54 with V600E mutation and 6/20 with non-V600E mutation). All of them received radical surgery and 10/15 received adjuvant chemotherapy. The median disease-free survival (DFS) was 15.3 months (3.0–63.9 months). No statistical difference was found between V600E/non-V600E patients (14.0 vs. 15.3 m, respectively, *p* = 0.257). However, Non-V600E mutant type at early stage showed better OS than V600E mutant type (not reached vs. 26.1 m, respectively, *p* = 0.05).

**Figure 4:**
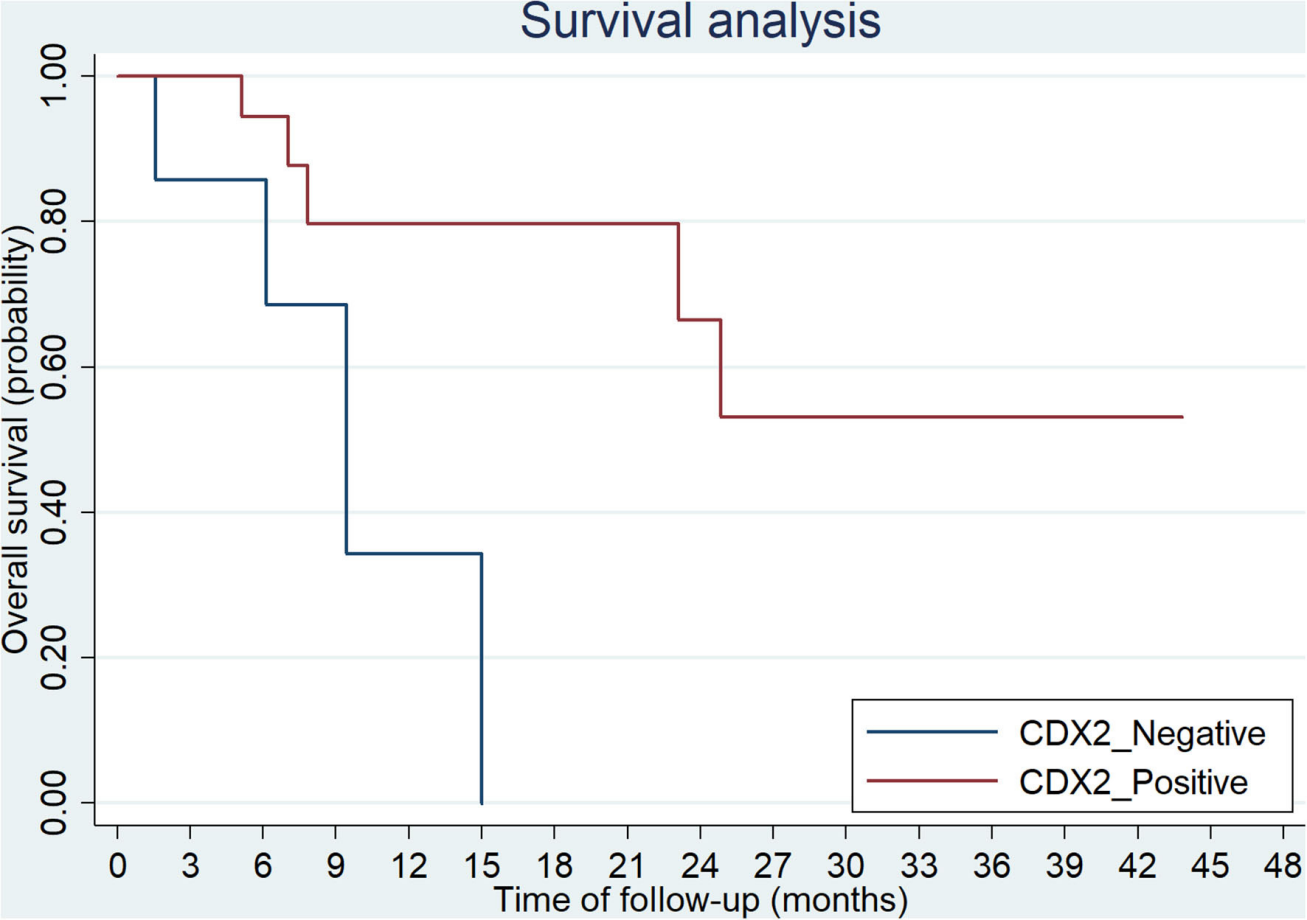
Overall survival of CRC patients with negative and positive CDX2 expression.

The overall survival after recurrence or metastasis was 18.9 months in *BRAF*^V600E^ group and not reached in *BRAF*^non−V600E^ group (*p* = 0.051). Multivariate analysis was showed in Table 4 and *BRAF*^V600E^ was an independent prognostic factor for survival. The univariate analysis showed that only CDX2 expression was related with prognosis of *BRAF*^V600E^ mutation patients, while gender, age, tumor location, tumor mutational burden (TMB) level and TNM stage were not (Table 5). Patients with negative CDX2 expression have worse outcome compared to those with positive CDX2 (mOS: 9.4 months vs. not reached, *p* = 0.016, Figure 4).

**Table 4:** Multivariate analysis for patients with *BRAF* mutated colorectal cancer.

**Characteristics**	***N***	**Overall survival**		
		**HR**	**95% CI**	***P***
Mutational status				
BRAF V600E	54	1		
BRAF non-V600E	20	0.34	0.14–0.84	0.019
Age				
<49 year	32	1		
≥49 year	42	1.98	0.87–4.48	0.102
Gender				
Male	45	1		
Female	29	1.28	0.61–2.69	0.523
Primary tumor site				
Right colon	26	1		
Left colon/rectum	48	1.38	0.61–3.12	0.436
Stage				
I, II, III	15	1		
IV	59	1.75	0.73–4.21	0.212

**Table 5:** Survival analysis for patients with *BRAF*^V600E^ mutated colorectal cancer.

	**mOS (months)**	***P*-value**
Gender		
Male	24.7	
Female	23.1	0.8116
Age		
<49	24.8	
>48	23.0	0.4016
Location		
Right-sided colon	26.1	
Left-sided colon	24.8	
Rectum	22.4	0.6703
TMB (Mut/Mb)		
<7.2	29.4	
>7.1	NA	0.5491
CDX2		
Negative	9.4	
Positive	NA	0.016
TNM stage		
II	NA	
III	23.1	
IV	22.4	0.3134

There were 3 *BRAF*^V600E^ patients who were alive for more than 40 months. One was a 66-year old male diagnosed as at stage II in 2013, who received radical surgery. The immunohistochemistry of primary tumor showed CDX2 positive and dMMR (MSH2 deficient). He got single lung metastasis after 5 years and received resection of the metastatic tumor and oral S1 as chemotherapy. He had multiple brain metastasis and received local radiotherapy in 2019. This patient was still in the follow-up. The other two patients were diagnosed at stage IV with pMMR and unknown CDX2 status. One got tumor located in rectum, with concurrent lung and peritoneum metastasis. The other one got left-sided colon cancer with peritoneum metastasis. Both patients received XELOX as first-line treatment and bevacizumab plus FOLFIRI regimen as second-line treatment.

## Discussion

It has been reported that *BRAF* mutated CRC patients have specific clinical, pathological and molecular characteristics, compared to patients with wild-type *BRAF* (14). Clinically, *BRAF* mutated CRCs are more often seen in elderly women, located in right-sided colon, and accompany with peritoneal and/or distant lymph node metastasis (4, 24). Regarding to pathological features, *BRAF*^V600E^ mutated CRCs are characterized by mucinous components, poor differentiation and highly aggressive behavior (14). In addition, *BRAF*^V600E^ mutation is associated with MSI-H/dMMR status, and mutually exclusive with RAS mutations (25–27). However, few studies have described the clinical, pathological and molecular features of non-V600E mutations. We tried to summarize the similarities and differences between V600E and non-V600E mutations in our single institution in China. The frequency of non-V600E mutations was 27% (20/74) in our study, similar with the rates reported in other literatures (15). Patients with non-V600E mutations showed no difference in gender, family history, metastatic sites, CDX2 status or TMB level compared with patients with V600E mutation. Regarding primary tumor sidedness, some studies demonstrated that CRC with non-V600E mutations might be more often on left side, but others found no relation between sidedness and non-V600E mutations (15, 16, 28). In this study, we found most of non-V600E mutated CRC located on left colon and rectum, but due to the small sample size, the difference was not statistically significant. Besides, we found 4 cases (4/20, 20%) with concomitant presence of both *BRAF* non-V600E and *RAS* mutations, though *BRAF* mutation was thought to be mutually exclusive with *RAS* mutations. Jones et al. also reported that patients with non-V600E mutant CRC were more likely with concomitant *RAS* mutation (15). According to previous literatures, though some subtypes of *BRAF* mutants had impaired or no kinase activity, they might retain oncogenic function by co-expression with other mutations, such as *RAS/EGFR* mutations (29). In fact, some researchers have identified *BRAF* mutations as three classes according to their acting pattern and *RAS* dependency: class 1 (V600 mutations) is activated monomers when *RAS* activity is low; class 2 (codon 464, 469, 597 and 601) acts as a *RAS*-independent dimer; class 3 (codon 287, 459, 466, 467, 469, 581, 594, 595, and 596) acts as a dimer with impaired kinase activity, so the oncogenic potential is *RAS*-dependent (29, 30). This may explain the concomitant presence of *BRAF* non-V600E and *RAS* mutations in some cases.

It has been broadly demonstrated that *BRAF*^V600E^ mutation is associated with poor prognosis of CRC patients regardless of stage (25, 31). According to our analysis, patients with non-V600E mutations had better OS than those with V600E mutation (*p* = 0.0239), especially for patients diagnosed at early stage. Shimada et al. reported V600E mutant type showed poorer OS than non-V600E mutant type after R0 resection (*p* = 0.038), which was consistent with our result. However, the prognostic value of non-V600E mutations is still controversial due to limited clinical data of this subgroup. Cremolini et al. found that some subtypes of non-V600E mutations (codon 594 and 596) might indicate a favorable outcome (28). More recently, Jones et al. reported a longer OS in patients with *BRAF* non-V600 mutations (60.7 months), which not only exceeded the OS of 11.4 months for patients with *BRAF*^V600E^ mutation, but also the survival of 43.0 months for patients with wild-type *BRAF* gene (15). Besides, they explored if the kinase activity (which was discussed above) would influence the OS of *BRAF*^non−V600E^ mutant patients. It turned out there was no significant difference in OS for patients with activated vs. impaired kinase (*p* = 0.544) (15). Hence the non-V600E mutated CRC may be a totally different subtype of CRC regarding to prognostic value.

Though *BRAF*^V600E^ mutation was associated with poorer survival in CRC, it has been observed that some patients with *BRAF*^V600E^ mutation have a relatively poorer outcome than others. Loupakis et al. classified patients with *BRAF*^V600E^ mutation as three different prognostic groups according to ECOG score, CA19-9 and LDH level, grade of tumor, status of metastasis (lung, liver and lymph nodes) (32). Prognosis of patients with *BRAF*^V600E^ mutation could be related to MMR/MSI status, or some genetic events occurring in pathogenesis of CRC (29, 30, 33). Recently, it was reported that CDX2 might play a significant role in prognosis of CRC (34, 35). CDX2 is a transcription factor and a specific marker of differentiation of intestine, which could be used to identified tumors originating from intestine (36). Aasebo et al. reported that CDX2 expression, which accounts for 53% of patients with *BRAF* mutation in their study, was associated with much better prognosis (34). Our study also demonstrated that loss of CDX2 expression indicated worse survival in patients with *BRAF*^V600E^ mutation (*p* = 0.016). Therefore, the loss of CDX2 expression may define a subgroup of poor prognosis in CRC patients, especially those with *BRAF*^V600E^ mutation.

Though the prognostic value was widely discussed in many studies, the predictive role of *BRAF* mutation in CRC patients received chemotherapy or targeted therapy remains unclear. Some studies showed *BRAF*^V600E^ mutated patients had worse PFS during first-line chemotherapy (10, 37); on the contrary, other studies reported that *BRAF* mutation was not associated with PFS of first-line treatment (8, 11, 38). The ambiguous results might depend on the small number of patients enrolled in the studies. Due to the aggressive behavior observed in *BRAF*^V600E^ mutated CRC, intensive chemotherapy combined with targeted therapy was used in first-line treatment and proved to be effective (39). It has been reported that FOLFOXIRI plus bevacizumab showed an improved response rate and PFS compared to chemotherapy alone in *BRAF* mutated CRC (17). In our study, bevacizumab plus chemotherapy regimen had a better response rate and longer median PFS compared to chemotherapy alone in *BRAF*^V600E^ mutated CRC patients, though the statistical significance was not reached. The response rate of bevacizumab plus FOLFOXIRI was 54.5%, which was an inspiring result considering the aggressiveness of *BRAF*^V600E^ mutated CRC.

Regimens including specific inhibitors against BRAF mutation and other components of MAPK pathway were proved to be effective in second-line treatment. The phase II SWOG S1406 trial showed that combination of vemurafenib, irinotecan and cetuximab (the “VIC” regimen) for *BRAF*^V600E^ mutant, *RAS* wild-type mCRC had an improved PFS compared with irinotecan plus cetuximab regimen (4.4 vs. 2.0 months) (18). Recently, the phase III BEACON trial proved an advantage of response rate and overall survival for combination of the BRAF inhibitor (encorafenib), MEK inhibitor (binimetinib) and cetuximab (20). Since MEK inhibitor was not available in China, we recorded only eight patients receiving the VIC regimen during second-line treatment. The ORR was 12.5% (1/8) and PFS was 2.9 months. The potential predictive value of different *BRAF* subtypes was less explored. Our studies showed that the subtypes of BRAF mutations had no significant impact on PFS during first-line or second-line treatment (*p* = 0.51 and 0.30, respectively).

There are some limitations of our study. First, it is a retrospective study and patients are from a single institution; hence selection bias inevitably exists. Most patients in this study were diagnosed at advanced stage, so the frequency of BRAF mutation in early staged CRC might be underestimated and its prognostic and predictive value is not clear. Second, given the rareness of BRAF mutation, especially non-V600E mutation, the sample size is too small to summarize the whole picture of CRC patients with BRAF mutation. Third, we were limited by lack of complete follow-up and treatment information for some patients.

## Conclusion

In summary, the clinical and pathological features and outcomes of BRAF mutated CRC patients are heterogeneous. While *BRAF*^V600E^ mutation is related with poor prognosis, non-V600E mutations define a subgroup of CRC patients with better outcome. Besides, some molecular basis like CDX2 status may affect the prognosis. So, it could be valuable to further classify BRAF mutated CRCs according to their molecular basis. The predictive value of BRAF mutation in CRC is still controversial; combination of different therapies may have better response compared to traditional chemotherapy. More efforts are needed to explore the molecular mechanism of BRAF mutation.

## Data Availability Statement

The data from this study can be found at the following link: http://download.omicsbio.info/files/BRAF_mut/.

## Ethics Statement

The studies involving human participants were reviewed and approved by ethics committee of Sun Yat-sen University Cancer Center. The patients provided written informed consent to participate in this study.

## Author Contributions

M-ZQ, F-HW, and R-HX: study design. W-LG and M-ZQ: literature search and writing. W-LG, M-ZQ, L-QY, YJ, Z-QW, Y-HL, F-HW, and R-HX: data collecting. C-YH: gene mutations tested. W-LG, M-ZQ, and L-QY: data analysis, figure, and tables. All authors contributed to the article and approved the submitted version.

## Conflict of Interest

The authors declare that the research was conducted in the absence of any commercial or financial relationships that could be construed as a potential conflict of interest.

## References

[1] Bray F Ferlay J Soerjomataram I Siegel RL Torre LA Jemal A. Global cancer statistics 2018: GLOBOCAN estimates of incidence and mortality worldwide for 36 cancers in 185 countries. CA Cancer J Clin. (2018) 68:394–424. 10.3322/caac.21492 30207593

[2] Sepulveda AR Hamilton SR Allegra CJ Greody W Cushman-Vokoun AM Funkhouser WK. Molecular biomarkers for the evaluation of colorectal cancer: guideline summary from the American society for clinical pathology, college of American pathologists, association for molecular pathology, and American society of clinical oncology. J Oncol Pract. (2017) 13:333–7. 10.1200/JOP.2017.022152 28350513

[3] Kim EK Choi EJ. Compromised MAPK signaling in human diseases: an update. Arch Toxicol. (2015) 89:867–82. 10.1007/s00204-015-1472-2 25690731

[4] Chen D Huang JF Liu K Zhang LQ Yang Z Chuai ZR. BRAFV600E mutation and its association with clinicopathological features of colorectal cancer: a systematic review and meta-analysis. PLoS ONE. (2014) 9:e90607. 10.1371/journal.pone.0090607 24594804 PMC3940924

[5] Chen D Zhang LQ Huang JF Liu K Chuai ZR Yang Z. BRAF mutations in patients with non-small cell lung cancer: a systematic review and meta-analysis. PLoS ONE. (2014) 9:e101354. 10.1371/journal.pone.0101354 24979348 PMC4076330

[6] Kim TH Park YJ Lim JA Ahn HY Lee EK Lee YJ. The association of the BRAF(V600E) mutation with prognostic factors and poor clinical outcome in papillary thyroid cancer: a meta-analysis. Cancer. (2012) 118:1764–73. 10.1002/cncr.26500 21882184

[7] Sanchez JN Wang T Cohen MS. BRAF and MEK inhibitors: use and resistance in BRAF-mutated cancers. Drugs. (2018) 78:549–66. 10.1007/s40265-018-0884-8 29488071 PMC6080616

[8] Seligmann JF Fisher D Smith CG Richman SD Elliott F Brown S. Investigating the poor outcomes of BRAF-mutant advanced colorectal cancer: analysis from 2530 patients in randomised clinical trials. Ann Oncol. (2017) 28:562–8. 10.1093/annonc/mdw645 27993800

[9] Tiacci E Trifonov V Schiavoni G Holmes A Kern W Martelli MP. BRAF mutations in hairy-cell leukemia. N Engl J Med. (2011) 364:2305–15. 10.1056/NEJMoa1014209 21663470 PMC3689585

[10] Tol J Nagtegaal ID Punt CJ. BRAF mutation in metastatic colorectal cancer. N Engl J Med. (2009) 361:98–9. 10.1056/NEJMc0904160 19571295

[11] Tie J Gibbs P Lipton L Christie M Jorissen RN Burgess AW. Optimizing targeted therapeutic development: analysis of a colorectal cancer patient population with the BRAF(V600E) mutation. Int J Cancer. (2011) 128:2075–84. 10.1002/ijc.25555 20635392

[12] Ye ZL Qiu MZ Tang T Wang F Zhou YX Lei MJ. Gene mutation profiling in Chinese colorectal cancer patients and its association with clinicopathological characteristics and prognosis. Cancer Med. (2020) 9:745–56. 10.1002/cam4.2727 31782259 PMC6970031

[13] Wan PT Garnett MJ Roe SM Lee S Niculescu-Duvaz D Good VM. Mechanism of activation of the RAF-ERK signaling pathway by oncogenic mutations of B-RAF. Cell. (2004) 116:855–67. 10.1016/S0092-8674(04)00215-6 15035987

[14] Fanelli GN Dal Pozzo CA Depetris I Schirripa M Brignola S Biason P. The heterogeneous clinical and pathological landscapes of metastatic Braf-mutated colorectal cancer. Cancer Cell Int. (2020) 20:30. 10.1186/s12935-020-1117-2 32015690 PMC6990491

[15] Jones JC Renfro LA Al-Shamsi HO Schrock AB Rankin A Zhang BY. (Non-V600) BRAF mutations define a clinically distinct molecular subtype of metastatic colorectal cancer. J Clin Oncol. (2017). 35:2624–30. 10.1200/JCO.2016.71.4394 28486044 PMC5549454

[16] Shinozaki E Yoshino T Yamazaki K Muro K Yamaguchi K Nishina T. Clinical significance of BRAF non-V600E mutations on the therapeutic effects of anti-EGFR monoclonal antibody treatment in patients with pretreated metastatic colorectal cancer: the biomarker research for anti-egfr monoclonal antibodies by comprehensive cancer genomics (BREAC) study. Br J Cancer. (2017) 117:1450–8. 10.1038/bjc.2017.308 28972961 PMC5680457

[17] Loupakis F Cremolini C Masi G Lonardi S Zagonel V Salvatore L. Initial therapy with FOLFOXIRI and bevacizumab for metastatic colorectal cancer. N Engl J Med. (2014) 371:1609–18. 10.1056/NEJMoa1403108 25337750

[18] Kopetz S McDonough SL Morris VK Lenz H-J Magliocco AM Atreya CE Randomized trial of irinotecan and cetuximab with or without vemurafenib in BRAF-mutant metastatic colorectal cancer (SWOG S1406) [abstract]. J Clin Oncol. (2017) 35:3505 10.1200/JCO.2017.35.15_suppl.3505 PMC8462593 33356422

[19] Hong DS Morris VK El Osta B Sorokin AV Janku F Fu S. Phase IB study of vemurafenib in combination with irinotecan and cetuximab in patients with metastatic colorectal cancer with BRAFV600E mutation. Cancer Discov. (2016) 6:1352–65. 10.1158/2159-8290.CD-16-0050 27729313 PMC5562357

[20] van Cutsem E Huijberts S Grothey A Yaeger R Cuyle PJ Elez E. Binimetinib, encorafenib, and cetuximab triplet therapy for patients with BRAF V600E-mutant metastatic colorectal cancer: safety lead-in results from the phase III BEACON colorectal cancer study. J Clin Oncol. (2019) 37:1460–9. 10.1200/JCO.18.02459 30892987 PMC7370699

[21] Qiu MZ He CY Lu SX Guan WL Wang F Wang XJ. Prospective observation: clinical utility of plasma Epstein-Barr virus DNA load in EBV-associated gastric carcinoma patients. Int J Cancer. (2020) 146:272–80. 10.1002/ijc.32490 31162842

[22] Zhang X Shi Y Song L Shen C Cai Q Zhang Z. Identification of mutations in patients with acquired pure red cell aplasia. Acta Biochim Biophys Sin. (2018) 50:685–92. 10.1093/abbs/gmy052 29767669

[23] Li M Zhao BR Liu SQ An J Deng PB Han-Zhang H. Mutational landscape and clonal diversity of pulmonary adenoid cystic carcinoma. Cancer Biol Ther. (2018) 19:898–903. 10.1080/15384047.2018.1480296 30067437 PMC6300342

[24] Jang MH Kim S Hwang DY Kim WY Lim SD Kim WS. BRAF-mutated colorectal cancer exhibits distinct clinicopathological features from wild-type BRAF-expressing cancer independent of the microsatellite instability status. J Korean Med Sci. (2017) 32:38–46. 10.3346/jkms.2017.32.1.38 27914130 PMC5143296

[25] Tran B Kopetz S Tie J Gibbs P Jiang ZQ Lieu CH. Impact of BRAF mutation and microsatellite instability on the pattern of metastatic spread and prognosis in metastatic colorectal cancer. Cancer. (2011) 117:4623–32. 10.1002/cncr.26086 21456008 PMC4257471

[26] Lochhead P Kuchiba A Imamura Y Liao X Yamauchi M Nishihara R. Microsatellite instability and BRAF mutation testing in colorectal cancer prognostication. J Natl Cancer Inst. (2013) 105:1151–6. 10.1093/jnci/djt173 23878352 PMC3735463

[27] Morkel M Riemer P Blaker H Sers C. Similar but different: distinct roles for KRAS and BRAF oncogenes in colorectal cancer development and therapy resistance. Oncotarget. (2015) 6:20785–800. 10.18632/oncotarget.4750 26299805 PMC4673229

[28] Cremolini C Di Bartolomeo M Amatu A Antoniotti C Moretto R Berenato R. BRAF codons 594 and 596 mutations identify a new molecular subtype of metastatic colorectal cancer at favorable prognosis. Ann Oncol. (2015) 26:2092–7. 10.1093/annonc/mdv290 26153495

[29] Yao Z Yaeger R Rodrik-Outmezguine VS Tao A Torres NM Chang MT. Tumours with class 3 BRAF mutants are sensitive to the inhibition of activated RAS. Nature. (2017) 548:234–8. 10.1038/nature23291 28783719 PMC5648058

[30] Yao Z Torres Neilawattie M Tao A Gao Y Luo L Li Q. BRAF mutants evade ERK-dependent feedback by different mechanisms that determine their sensitivity to pharmacologic inhibition. Cancer Cell. (2015) 28:370–83. 10.1016/j.ccell.2015.08.001 26343582 PMC4894664

[31] Van Cutsem E Köhne C-H Láng I Folprecht G Nowacki MP Cascinu S. Cetuximab plus irinotecan, fluorouracil, and leucovorin as first-line treatment for metastatic colorectal cancer: updated analysis of overall survival according to tumor KRAS and BRAF mutation status. J Clin Oncol. (2011) 29:2011–9. 10.1200/JCO.2010.33.5091 21502544

[32] Loupakis F Intini R Cremolini C Orlandi A Sartore-Bianchi A Pietrantonio F. A validated prognostic classifier for BRAF-mutated metastatic colorectal cancer: the ‘BRAF BeCool’ study. Eur J Cancer. (2019) 118:121–30. 10.1016/j.ejca.2019.06.008 31330487

[33] Samowitz WS Sweeney C Herrick J Albertsen H Levin TR Murtaugh MA. Poor survival associated with the BRAF V600E mutation in microsatellite-stable colon cancers. Cancer Res. (2005) 65:6063–9. 10.1158/0008-5472.CAN-05-0404 16024606

[34] Aasebo K Dragomir A Sundstrom M Mezheyeuski A Edqvist PH Eide GE. CDX2: a prognostic marker in metastatic colorectal cancer defining a better BRAF mutated and a worse KRAS mutated subgroup. Front Oncol. (2020) 10:8. 10.3389/fonc.2020.00008 32117703 PMC7026487

[35] Loupakis F Biason P Prete AA Cremolini C Pietrantonio F Pella N. CK7 and consensus molecular subtypes as major prognosticators in (V600E)BRAF mutated metastatic colorectal cancer. Br J Cancer. (2019) 121:593–9. 10.1038/s41416-019-0560-0 31474758 PMC6889398

[36] Werling RW Yaziji H Bacchi CE Gown AM. CDX2, a highly sensitive and specific marker of adenocarcinomas of intestinal origin: an immunohistochemical survey of 476 primary and metastatic carcinomas. Am J Surg Pathol. (2003) 27:303–10. 10.1097/00000478-200303000-00003 12604886

[37] Souglakos J Philips J Wang R Marwah S Silver M Tzardi M. Prognostic and predictive value of common mutations for treatment response and survival in patients with metastatic colorectal cancer. Br J Cancer. (2009) 101:465–72. 10.1038/sj.bjc.6605164 19603024 PMC2720232

[38] Kayhanian H Goode E Sclafani F Ang JE Gerlinger M Gonzalez de Castro D. Treatment and survival outcome of BRAF-mutated metastatic colorectal cancer: a retrospective matched case-control study. Clin Colorectal Cancer. (2018) 17:e69–76. 10.1016/j.clcc.2017.10.006 29129559

[39] Falcone A Ricci S Brunetti I Pfanner E Allegrini G Barbara C. Phase III trial of infusional fluorouracil, leucovorin, oxaliplatin, and irinotecan (FOLFOXIRI) compared with infusional fluorouracil, leucovorin, and irinotecan (FOLFIRI) as first-line treatment for metastatic colorectal cancer: the Gruppo oncologico nord ovest. J Clin Oncol. (2007) 25:1670–6. 10.1200/JCO.2006.09.0928 17470860

